# Supplementary material for: The Effect of Rainfall and Temperature Patterns on Childhood Linear Growth in the Tropics: Systematic Review and Meta-Analysis
Source: Int J Environ Res Public Health. 2024 Sep 25;21(10):1269. doi: 10.3390/ijerph21101269 (PMC11506850; doi:10.3390/ijerph21101269)
Supplement: Supplementary file 1 [file ijerph-21-01269-s001.zip › ijerph-3170477-supplementary.pdf]

Supplementary materials

Supplementary S1: Search strategy and terms.

1. Pubmed

(((((((("climate variability"[All Fields]) OR ("weather"[All Fields])) OR ("rainfall"[All Fields])) OR ("temperature"[All Fields])) OR ("precipitation"[All Fields])) OR ("seasonality"[All Fields])) OR ("drought"[All Fields])) AND ((("stunting"[All Fields]) OR ("growth disorder"[All Fields])) OR ("undernutrition"[All Fields]))) AND (("angola"[All Fields]) OR ("antigua and barbuda"[All Fields])) OR ("aruba"[All Fields])) OR ("barbados"[All Fields])) OR ("belize"[All Fields])) OR ("benin"[All Fields])) OR ("british virgin islands"[All Fields])) OR ("burkina faso"[All Fields])) OR ("burundi"[All Fields])) OR ("cambodia"[All Fields])) OR ("cameroon"[All Fields])) OR ("cape verde"[All Fields])) OR ("central african republic"[All Fields])) OR ("chad"[All Fields])) OR ("colombia"[All Fields])) OR ("comoros"[All Fields])) OR ("costa rica"[All Fields])) OR ("cuba"[All Fields])) OR ("djibouti"[All Fields])) OR ("dominica"[All Fields])) OR ("dominican republic"[All Fields])) OR ("democratic republic of congo"[All Fields])) OR ("ecuador"[All Fields])) OR ("el salvador"[All Fields])) OR ("equatorial guinea"[All Fields])) OR ("eritrea"[All Fields])) OR ("ethiopia"[All Fields])) OR ("fiji"[All Fields])) OR ("french guiana"[All Fields])) OR ("gabon"[All Fields])) OR ("gambia"[All Fields])) OR ("ghana"[All Fields])) OR ("grenada"[All Fields])) OR ("guadeloupe"[All Fields])) OR ("guatemala"[All Fields])) OR ("guinea"[All Fields])) OR ("guinea bissau"[All Fields])) OR ("guyana"[All Fields])) OR ("haiti"[All Fields])) OR ("honduras"[All Fields])) OR ("indonesia"[All Fields])) OR ("ivory coast"[All Fields])) OR ("jamaica"[All Fields])) OR ("kenya"[All Fields])) OR ("kiribati"[All Fields])) OR ("laos"[All Fields])) OR ("liberia"[All Fields])) OR ("malawi"[All Fields])) OR ("malaysia"[All Fields])) OR ("maldives"[All Fields])) OR ("mali"[All Fields])) OR ("marshall islands"[All Fields])) OR ("martinique"[All Fields])) OR ("mauritania"[All Fields])) OR ("mauritius"[All Fields])) OR ("micronesia"[All Fields])) OR ("nauru"[All Fields])) OR ("nicaragua"[All Fields])) OR ("niger"[All Fields])) OR ("nigeria"[All Fields])) OR ("palau"[All Fields])) OR ("panama"[All Fields])) OR ("papua new guinea"[All Fields])) OR ("peru"[All Fields])) OR ("philippines"[All Fields])) OR ("republic of the congo"[All Fields])) OR ("rwanda"[All Fields])) OR ("saint kitts and nevis"[All Fields])) OR ("saint lucia"[All Fields])) OR ("saint vincent"[All Fields])) OR ("the grenadines"[All Fields])) OR ("samoa"[All Fields])) OR ("sao tome and principe"[All Fields])) OR ("senegal"[All Fields])) OR ("seychelles"[All Fields])) OR ("sierra leone"[All Fields])) OR ("singapore"[All Fields])) OR ("solomon islands"[All Fields])) OR ("somalia"[All Fields])) OR ("south sudan"[All Fields])) OR ("sri lanka"[All Fields])) OR ("sudan"[All Fields])) OR ("suriname"[All Fields])) OR ("tanzania"[All Fields])) OR ("thailand"[All Fields])) OR ("togo"[All Fields])) OR ("tonga"[All Fields])) OR ("trinidad and tobago"[All Fields])) OR ("tuvalu"[All Fields])) OR ("uganda"[All Fields])) OR ("united states virgin islands"[All Fields])) OR ("vanuatu"[All Fields])) OR ("venezuela"[All Fields])) OR ("vietnam"[All Fields])) OR ("yemen"[All Fields])) OR ("zambia"[All Fields]))

2. EMBASE

('climate change' OR 'extreme weather' OR rain OR temperature OR heat OR precipitation) AND (stunting OR 'growth disorder' OR 'height for age z score' OR 'malnutrition assessment' OR 'length for age') AND (angola OR 'antigua and barbuda' OR aruba OR barbados OR belize OR benin OR 'virgin islands (british)' OR 'burkina faso' OR burundi OR cambodia OR cameroon OR 'cape verde' OR 'central african republic' OR chad OR colombia OR comoros OR 'costa rica' OR cuba OR djibouti OR 'dominican republic' OR 'democratic republic congo' OR ecuador OR 'el salvador' OR 'equatorial guinea' OR eritrea OR ethiopia OR

fiji OR 'french guiana' OR gabon OR gambia OR ghana OR grenada OR guadeloupe OR guatemala OR guinea OR 'guinea bissau' OR guyana OR haiti OR honduras OR indonesia OR 'cote d`ivoire' OR jamaica OR kenya OR kiribati OR laos OR liberia OR malawi OR malaysia OR maldives OR mali OR 'marshall islands' OR martinique OR mauritania OR mauritius OR 'federated states of micronesia' OR nauru OR nicaragua OR niger OR nigeria OR palau OR panama OR 'papua new guinea' OR peru OR philippines OR congo OR rwanda OR 'saint kitts and nevis' OR 'saint lucia' OR 'saint vincent and the grenadines' OR samoa OR 'sao tome and principe' OR senegal OR seychelles OR 'sierra leone' OR singapore OR 'solomon islands' OR somalia OR 'south sudan' OR 'sri lanka' OR sudan OR suriname OR tanzania OR thailand OR togo OR tonga OR 'trinidad and tobago' OR tuvalu OR uganda OR 'virgin islands (u.s.)' OR vanuatu OR venezuela OR 'viet nam' OR yemen OR zambia)

3. Scopus

(TITLE-ABS-KEY (climate AND variability OR weather OR rainfall OR temperature OR precipitation OR season* OR drought) AND PUBYEAR > 2003 AND PUBYEAR < 2025) AND (TITLE-ABS-KEY (stunting OR "height-for-age" OR "length-for-age" OR haz OR short* OR undernutrition OR "growth disorder") AND PUBYEAR > 2003 AND PUBYEAR < 2025) AND (TITLE-ABS-KEY ("under five" OR children OR "infant and young child*" OR "0-59 month") AND PUBYEAR > 2003 AND PUBYEAR < 2025) AND (TITLE-ABS-KEY (angola OR "Antigua and Barbuda" OR aruba OR barbados OR belize OR benin OR "British Virgin Islands" OR "Burkina Faso" OR burundi OR cambodia OR cameroon OR "Cape Verde" OR "Central African Republic" OR chad OR colombia OR comoros OR "Costa Rica" OR cuba OR djibouti OR dominica OR "Dominican Republic" OR "Democratic Republic of Congo" OR ecuador OR salvador OR "Equatorial Guinea" OR eritrea OR ethiopia OR fiji OR "French Guiana" OR gabon OR gambia OR ghana OR grenada OR guadeloupe OR guatemala OR guinea OR "Guinea-Bissau" OR guyana OR haiti OR honduras OR indonesia OR "Ivory Coast" OR jamaica OR kenya OR kiribati OR laos OR liberia OR malawi OR malaysia OR maldives OR mali OR "Marshall Islands" OR martinique OR mauritania OR mauritius OR micronesia OR nauru OR nicaragua OR niger OR nigeria OR palau OR panama OR "Papua New Guinea" OR peru OR philippines OR "Republic of the Congo" OR rwanda OR "Saint Kitts and Nevis" OR "Saint Lucia" OR "Saint Vincent and the Grenadines" OR samoa OR "Sao Tome and Principe" OR senegal OR seychelles OR "Sierra Leone" OR singapore OR "Solomon Islands " OR somalia OR "South Sudan" OR "Sri Lanka" OR sudan OR suriname OR tanzania OR thailand OR togo OR tonga OR "Trinidad and Tobago" OR tuvalu OR uganda OR "United States Virgin Islands" OR vanuatu OR venezuela OR vietnam OR yemen OR zambia) AND PUBYEAR > 2003 AND PUBYEAR < 2025) AND (LIMIT-TO (LANGUAGE , "English")) AND (LIMIT-TO (DOCTYPE , "ar"))

4. Cochrane library

"climate variability" OR weather OR rainfall OR temperature OR precipitation OR Season* OR flood OR drought
stunting OR "growth disorders" OR undernutrition
"under five" OR children OR "infant and young children"
angola OR "antigua and barbuda" OR aruba OR barbados OR belize OR benin OR "virgin islands (british)" OR "burkina faso" OR burundi OR cambodia OR cameroon OR "cape verde" OR "central african republic" OR chad OR colombia OR comoros OR "costa rica" OR cuba OR djibouti OR "dominican republic" OR "democratic republic congo" OR ecuador OR "el salvador" OR "equatorial guinea" OR eritrea OR ethiopia OR fiji OR "french guiana" OR gabon OR gambia OR ghana OR grenada OR guadeloupe OR guatemala OR guinea OR "guinea bissau" OR guyana OR haiti OR honduras OR indonesia OR "cote d`ivoire" OR jamaica OR kenya OR kiribati OR laos OR liberia OR malawi OR malaysia OR maldives OR mali OR "marshall

islands" OR martinique OR mauritania OR mauritius OR "federated states of micronesia" OR nauru OR nicaragua OR niger OR nigeria OR palau OR panama OR "papua new guinea" OR peru OR philippines OR congo OR rwanda OR "saint kitts and nevis" OR "saint lucia" OR "saint vincent and the grenadines" OR samoa OR "sao tome and principe" OR senegal OR seychelles OR "sierra leone" OR singapore OR "solomon islands" OR somalia OR "south sudan" OR "sri lanka" OR sudan OR suriname OR tanzania OR thailand OR togo OR tonga OR "trinidad and tobago" OR tuvalu OR uganda OR "virgin islands (u.s.)" OR vanuatu OR venezuela OR "viet nam" OR yemen OR Zambia
#1 AND #2 AND #3 AND #4

5. Science Direct

effect of weather(temperature, rainfall, precipitation, drought, flood) anomalies on stunting from 2004 to 2024

6. Mednar and worldwide science

effect of weather(temperature, rainfall, precipitation, drought, flood) anomalies on stunting

Table S2: Quality assessment using JBI quality criteria

Table 1: Evaluation of studies as per JBI quality criteria

Author (year)	1. Criteria for inclusion in the sample clearly defined	2. Study subjects and the setting described in detail	3. Exposure measured in a valid and reliable way	4. Objective, standard criteria used for measurement of the condition	5. Confounding factors identified	6. Strategic to deal with confounding factors stated	7. Outcomes measured in a valid and reliable way	8. Appropriate statistical analysis used	Overall	Decision
Abiona (2024)	Yes	Yes	Yes	Yes	Yes	Yes	Yes	Yes	8	Low risk of bias
Aheto and Dagne (2021)	Yes	Yes	Yes	Yes	Yes	Yes	Yes	Yes	8	Low risk of bias
Amegbor et al. (2020)	Yes	Yes	Yes	Yes	Yes	Yes	Yes	Yes	8	Low risk of bias
Atalell et al. (2023)	Yes	Yes	Yes	Yes	Yes	Yes	Yes	Yes	8	Low risk of bias
Baffour et al. (2023)	Yes	Yes	Yes	Yes	Yes	Yes	Yes	Yes	8	Low risk of bias
Block et al. (2022)	Yes	Yes	Yes	Yes	Yes	Yes	Yes	Yes	8	Low risk of bias
Boyd (2023)	Yes	Yes	Yes	Yes	Yes	Yes	Yes	Yes	8	Low risk of bias
Cliffer et al. (2024)	Yes	Yes	Yes	Yes	Yes	Yes	Yes	Yes	8	Low risk of bias
Cooper et al. (2019)	Yes	Yes	Yes	Yes	Yes	Yes	Yes	Yes	8	Low risk of bias
Cooper et al. (2019)	Yes	Yes	Yes	Yes	Yes	Yes	Yes	Yes	8	Low risk of bias
Cornwell and Inder (2015)	Yes	Unclear	Yes	Yes	Yes	Yes	Yes	Yes	7	Low risk of bias
Davenport et al. (2017)	Yes	Yes	Yes	Yes	Yes	Yes	Yes	Yes	8	Low risk of bias
Epstein et al. (2019)	Yes	Yes	Yes	Yes	Yes	Yes	Yes	Yes	8	Low risk of bias
Grace et al. (2012)	Yes	Yes	Yes	Yes	Yes	Yes	Yes	Yes	8	Low risk of bias

Hagos et al. (2014)	Unclear	Yes	Yes	Yes	Yes	Yes	Yes	Yes	7	Low risk of bias
Hongoli and Hahn (2023)	Yes	Yes	Yes	Yes	Yes	Yes	Yes	Yes	8	Low risk of bias
Mank et al. (2021)	Yes	Yes	Yes	Yes	Yes	Yes	Yes	Yes	8	Low risk of bias
Mukabutera et al. (2016)	Yes	Yes	Yes	Yes	Yes	Yes	Yes	Yes	8	Low risk of bias
Ngwira (2020)	Yes	Yes	Yes	Yes	Yes	Yes	Yes	Yes	8	Low risk of bias
Nicholas et al. (2021)	Yes	Yes	Yes	Yes	Yes	Yes	Yes	Yes	8	Low risk of bias
Rabassa et al. (2014)	Yes	Yes	Yes	Yes	Yes	Yes	Yes	Yes	8	Low risk of bias
Rahut et al. (2024)	Yes	Yes	Yes	Yes	Yes	Yes	Yes	Yes	8	Low risk of bias
Randell et al. (2020)	Yes	Yes	Yes	Yes	Yes	Yes	Yes	Yes	8	Low risk of bias
Rojas et al. (2023)	Yes	Yes	Yes	Yes	Yes	Yes	Yes	Yes	8	Low risk of bias
Ssentongo et al. (2020)	Yes	Yes	Yes	Yes	Yes	Yes	Yes	Yes	8	Low risk of bias
Thiede and Gray (2020)	Yes	Yes	Yes	Yes	Yes	Yes	Yes	Yes	8	Low risk of bias
van der Merwe et al.(2022)	Yes	Yes	Yes	Yes	Yes	Yes	Yes	Yes	8	Low risk of bias
Yeboah et al. (2022)	Yes	Yes	Yes	Yes	Yes	Yes	Yes	Yes	8	Low risk of bias
Ayalew (2023)	Yes	Yes	Yes	Yes	Yes	Yes	Yes	Yes	8	Low risk of bias
Ahmed et al. (2023)	Yes	Yes	Yes	Yes	Yes	Yes	Yes	Yes	8	Low risk of bias
Jamali (2018)	Yes	Unclear	Yes	Yes	Yes	Yes	Yes	Yes	7	Low risk of bias
Blom et al. (2022)	Yes	Yes	Yes	Yes	Yes	Yes	Yes	Yes	8	Low risk of bias
Elayouty et al. (2022)	Yes	Yes	Yes	Yes	Yes	Yes	Yes	Yes	8	Low risk of bias

Injete et al. (2021)	Yes	Yes	Yes	Yes	Yes	Yes	Yes	Yes	8	Low risk of bias
Kinyoki et al. (2017)	Yes	Yes	Yes	Yes	Yes	Yes	Yes	Yes	8	Low risk of bias
Kinyoki et al. (2016)	Yes	Yes	Yes	Yes	Yes	Yes	Yes	Yes	8	Low risk of bias
Tanou et al. (2024)	Yes	Yes	Yes	Yes	Yes	Yes	Yes	Yes	8	Low risk of bias
Araujo et al. (2012)	Yes	Yes	Yes	Yes	Yes	Yes	Yes	Yes	8	Low risk of bias
Lopez-Carr et al. (2016)	Yes	Yes	Yes	Yes	Yes	Yes	Yes	Yes	8	Low risk of bias

Figure S3: Publication bias for rainfall and childhood linear growth

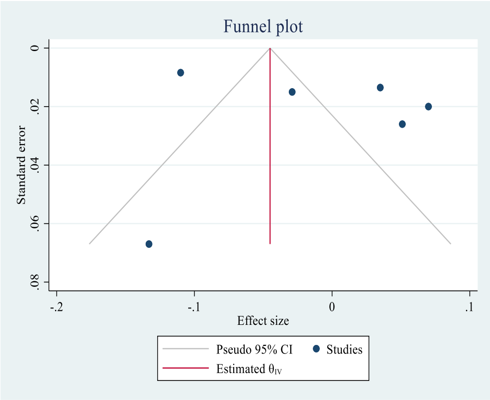

Figure 1: Funnel plot showing pooled effect size (β) of rainfall on childhood linear growth

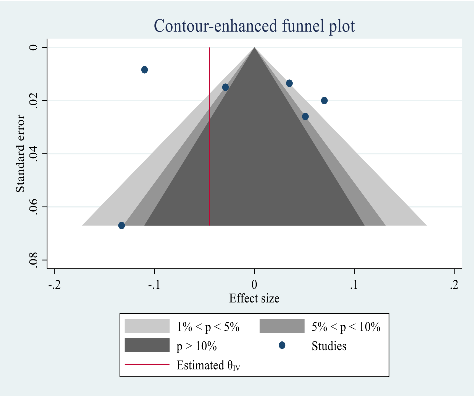

Figure 2: Contour enhanced funn el plot showing pooled effect size (β) of rainfall on childhood linear growth

Figure S4: Publication bias, Eggers test, and Nonparametric trim-and-fill analysis of publication bias for temperature and childhood linear growth

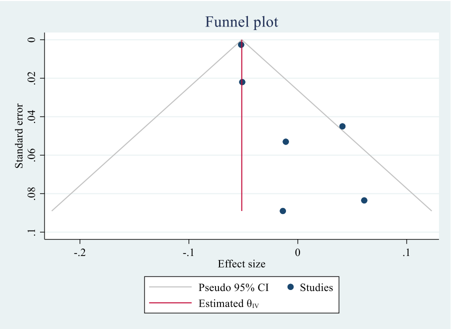

Figure 3: Funnel plot showing pooled effect size (*β*) of temperature on childhood linear growth

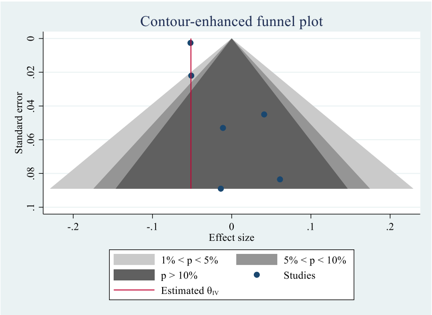

Figure 4: Contour -enhanced funnel plot showing pooled effect size (*β*) of temperature on childhood linear growth

Regression-based Egger test for small-study effects
Random-effects model
Method: DerSimonian-Laird

H0: beta1 = 0; no small-study effects

beta1 = 1.00
SE of beta1 = 0.478
z = 2.10
Prob > |z| = 0.0358

Nonparametric trim-and-fill analysis of publication bias
Linear estimator, imputing on the left

Iteration — Number of studies = 9
Model: Random-effects — observed = 6
Method: DerSimonian-Laird — imputed = 3

Pooling
Model: Random-effects
Method: DerSimonian-Laird

Studies	Effect Size	[95% Conf. Interval]	
Observed	-0.039	-0.065	-0.013
Observed + Imputed	-0.051	-0.082	-0.020

Figure S5: Publication bias for temperature and stunting

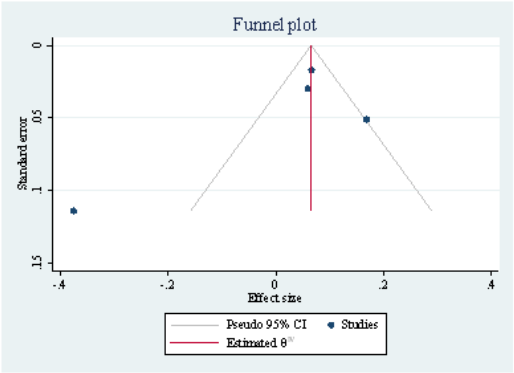

Figure 5: Funnel plot showing pooled effect size (β) of temperature on childhood linear growth failure

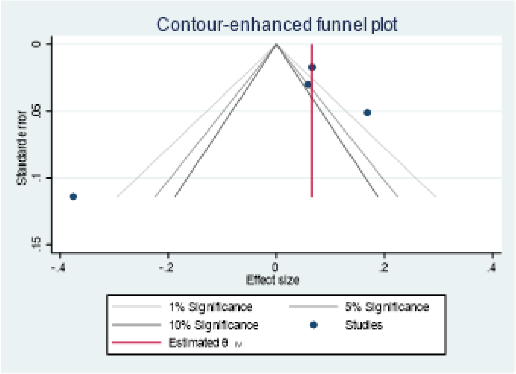

Figure 6: Contour-enhanced funnel plot showing pooled effect size (β) of temperature on childhood linear growth failure
